# Neonatal Immature Retroperitoneal Teratoma With Pancreatic Involvement and Intraoperative Findings Mimicking Fetus in Fetu

**DOI:** 10.7759/cureus.117703

**Published:** 2026-10-09

**Authors:** Paulina Estefanía Sánchez Román, Jimmy Israel Andrade Montesdeoca, Vicente Aníbal Salinas Salinas, Jorge Oliveros-Rivero

**Affiliations:** 1 Pediatric Surgery, Universidad Católica de Santiago de Guayaquil, Guayaquil, ECU; 2 Pediatric Surgery, Dr. Roberto Gilbert Children’s Hospital, Guayaquil, ECU; 3 Pediatric Surgery, Hospital General Monte Sinaí, Guayaquil, ECU

**Keywords:** abdominal mass, fetus in fetu, immature teratoma, neonate, pancreatic involvement, pediatric surgery, retroperitoneal teratoma

## Abstract

Retroperitoneal teratomas are uncommon pediatric germ cell tumors and are exceptional during the neonatal period. Their diagnosis and treatment are challenging because they may compress major abdominal vessels and involve adjacent organs. We report the case of a two-day-old male neonate who presented with progressive abdominal distension and a palpable abdominal mass. Ultrasonography and computed tomography demonstrated a 9 × 9 cm heterogeneous retroperitoneal lesion with solid, cystic, and calcified components, associated with displacement of bowel loops, compression of major vessels, and apparent pancreatic involvement. Exploratory laparotomy with tumor resection was performed. Intraoperatively, the lesion showed macroscopic structures resembling a hand and a foot, raising the differential diagnosis of fetus in fetu. The pancreatic tail was considered involved intraoperatively and was resected, while the pancreatic head and body were preserved. Histopathological examination confirmed a grade 3 extragonadal immature teratoma with no vertebral axis. The pathology report also described partial pancreatic tail involvement; however, the available pathology documentation did not further characterize the pancreatic-tumor interface or the pancreatic resection margin. The postoperative hospital course was favorable, although prolonged neonatal intensive care was required. This case highlights the importance of preoperative imaging, careful retroperitoneal dissection, vascular control, patient-specific clarification of distorted anatomy, and histopathological confirmation when evaluating complex neonatal abdominal masses.

## Introduction

Teratomas are germ cell tumors composed of tissues derived from the three embryonic germ layers and are classified according to their degree of histological maturation. Retroperitoneal teratomas represent an uncommon subgroup of pediatric germ cell tumors, and presentation during the neonatal period is particularly rare [1-6].

In neonates, a heterogeneous retroperitoneal mass containing solid, cystic, and calcified components raises a broad differential diagnosis that includes germ cell tumors, adrenal lesions, enteric duplications, congenital cystic malformations, and fetus in fetu. Published neonatal retroperitoneal teratoma reports and pediatric series remain limited, particularly for lesions with close pancreatic relationships and complex abdominal vascular anatomy [5,7-11].

Among the available reports cited here, detailed descriptions combining grade 3 histologic immaturity, pancreatic tail involvement requiring resection, complex relationships with major abdominal vessels, and intraoperative fetiform morphology are sparse [5,7-11]. Fetiform macroscopic features may also create diagnostic overlap between teratoma and fetus in fetu, requiring correlation among imaging, operative findings, and histopathology rather than reliance on gross appearance alone [12-15].

This report describes a neonatal grade 3 immature retroperitoneal teratoma requiring pancreatic tail resection, with complex vascular relationships and organized macroscopic structures resembling a hand and a foot. The case contributes a detailed clinicoradiologic, operative, and histopathologic description of this unusual combination and illustrates the importance of distinguishing intraoperative fetiform appearance from the definitive histopathologic diagnosis.

## Case presentation

A two-day-old male neonate was delivered at 39 weeks of gestation according to the Ballard assessment, with Apgar scores of 7 and 8 and a birth weight of 3,050 g. His 32-year-old mother reported five prenatal ultrasound examinations without identified fetal abnormalities. Progressive abdominal distension had been present since birth.

Physical examination revealed marked abdominal distension, visible collateral abdominal veins, hepatomegaly extending approximately 5 cm below the right costal margin, and a palpable mass measuring approximately 12 cm, predominantly occupying the left hemiabdomen. The abdominal circumference was 35 cm.

Initial laboratory investigations and age-specific reference intervals are presented in Table 1.

**Table 1:** Initial laboratory findings with age-specific reference intervals Laboratory values were obtained on postnatal day 2. Reference intervals are age-, assay-, and laboratory-dependent. Procalcitonin undergoes a physiological postnatal rise during the first 72 hours of life, whereas alpha-fetoprotein is physiologically elevated during the neonatal period and decreases progressively with age. AFP: alpha-fetoprotein; LDH: lactate dehydrogenase.

Laboratory investigation	Patient result	Age-specific reference interval
Leukocyte count	16.84 × 10⁹/L	9.0-30.0 × 10⁹/L [16]
Procalcitonin	4.05 ng/mL	0.00-20.00 ng/mL during the first 72 h of life [17]
Lactate dehydrogenase	597 U/L	125-735 U/L [18]
Alpha-fetoprotein	605,000 ng/mL	6,950-144,544 ng/mL at postnatal day 2 [19]

Ultrasound identified a 9 × 9 cm retroperitoneal mass with an estimated volume of 239 mL. The available color Doppler frame demonstrates a heterogeneous solid component, an internal echogenic focus compatible with calcification, and adjacent vascular flow (Figure 1). The complete ultrasound examination documented additional cystic components, pancreatic compression, and a close relationship with the major abdominal vessels.

**Figure 1:**
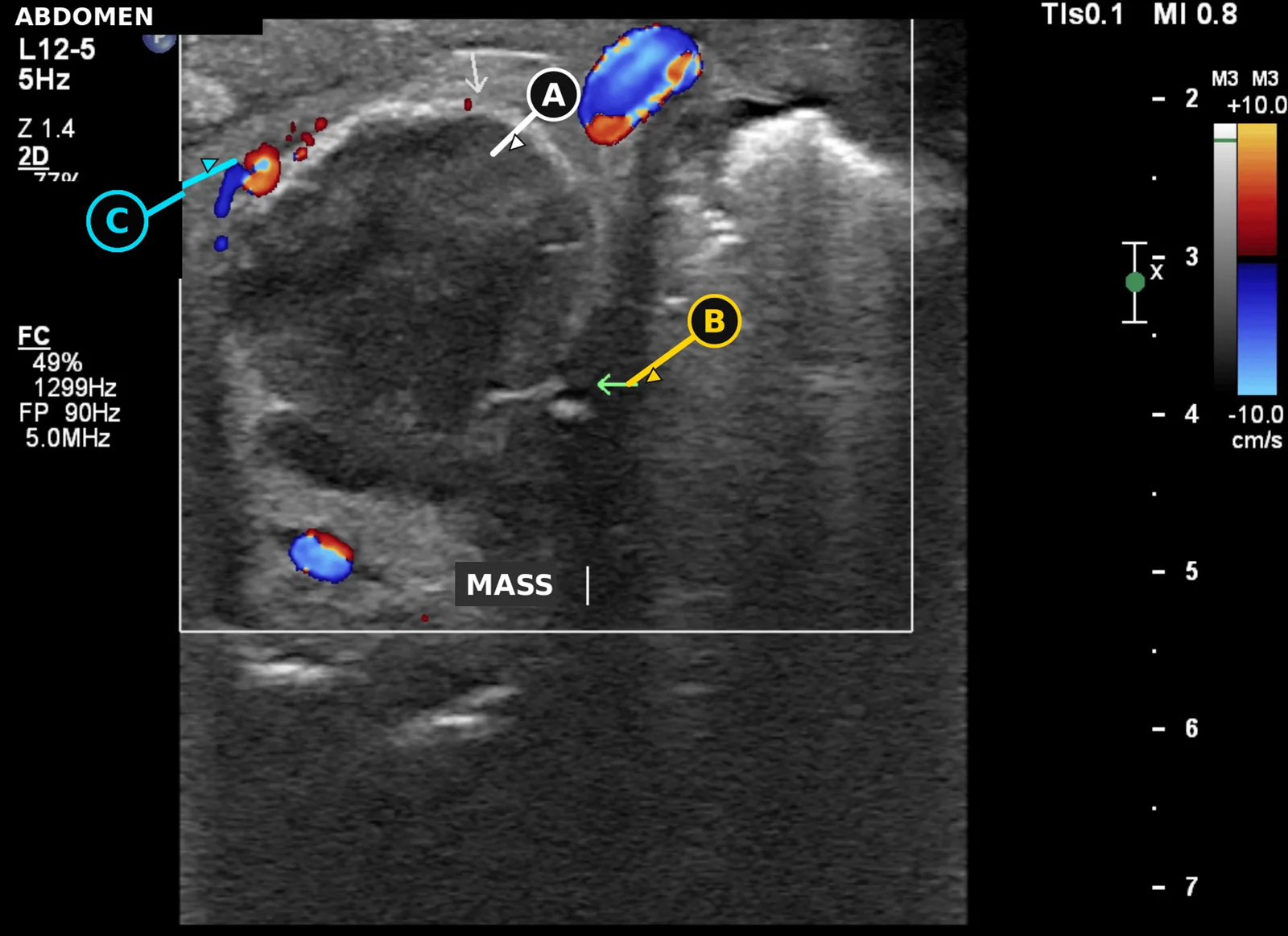
Color Doppler ultrasound of the retroperitoneal mass Color Doppler ultrasonography shows a large heterogeneous retroperitoneal mass. The white arrow (A) indicates the superior margin of its heterogeneous solid component; the yellow arrow (B) indicates an internal echogenic focus compatible with calcification; and the cyan arrow (C) indicates adjacent peripheral vascular flow.

Computed tomography confirmed a heterogeneous lesion with 28 Hounsfield unit solid areas, 10 Hounsfield unit fluid content, internal calcifications, and displacement of the intestinal loops (Figure 2).

**Figure 2:**
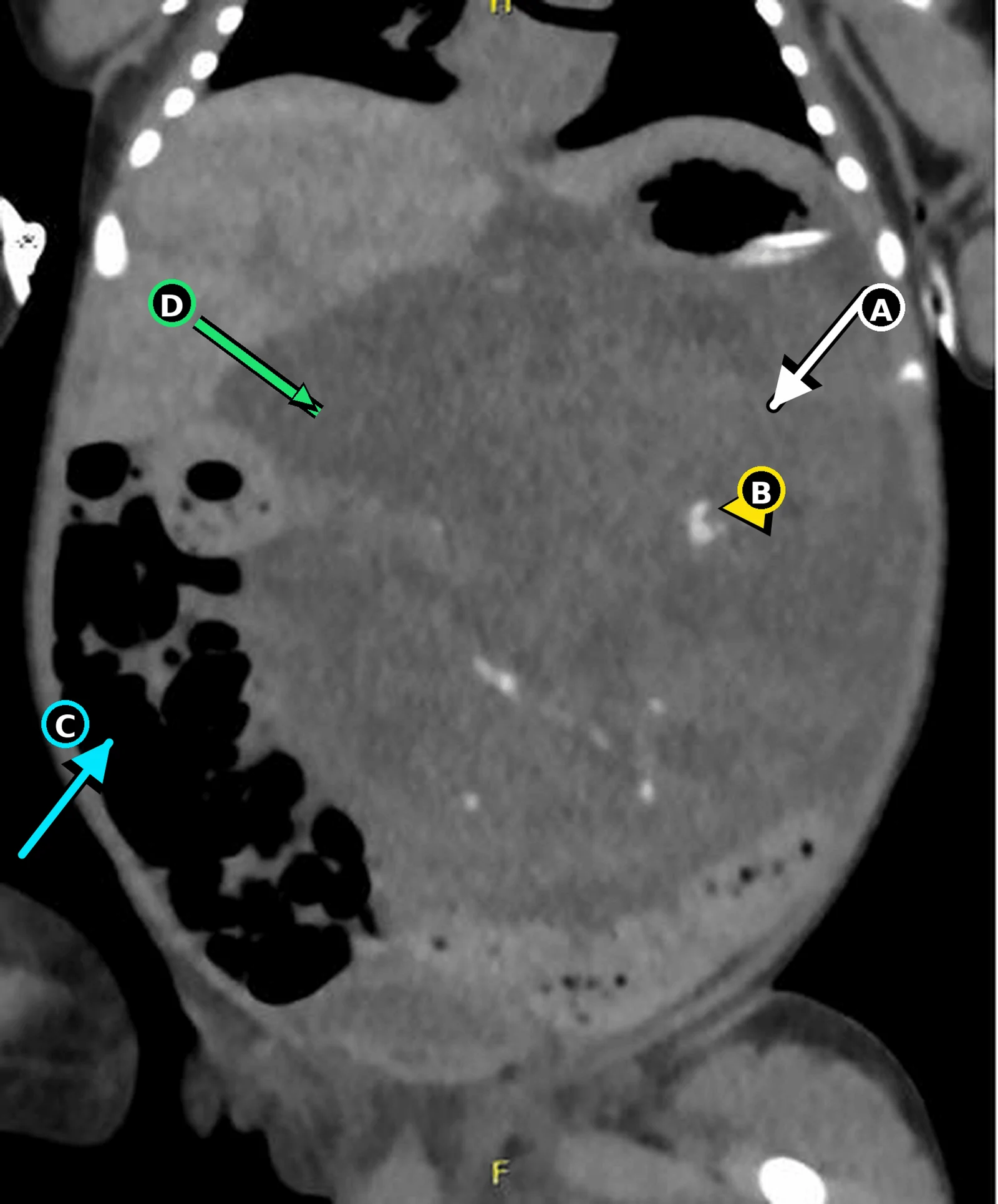
Contrast-enhanced computed tomography of the retroperitoneal mass Coronal contrast-enhanced computed tomography highlighting the key radiologic findings: (A) large heterogeneous retroperitoneal mass (white arrow); (B) internal calcified focus (yellow arrowhead); (C) displaced gas-filled intestinal loops (cyan arrow); and (D) low-attenuation fluid component within the mass (green arrow).

Given the presence of a large complex neonatal retroperitoneal tumor, its close relationship with the pancreas and major vascular structures, and the need for definitive diagnosis and treatment, exploratory laparotomy with tumor resection was performed once the patient was clinically suitable for surgery.

Intraoperatively, a multilobulated mass occupying the left hemiabdomen and extending into the retroperitoneum was identified (Figure 3). The lesion was closely related to the pancreatic body and tail and received arterial supply from vessels arising from the abdominal aorta, with venous drainage toward the inferior vena cava. The vascular pedicles were controlled with titanium clips and 4-0 braided silk sutures.

**Figure 3:**
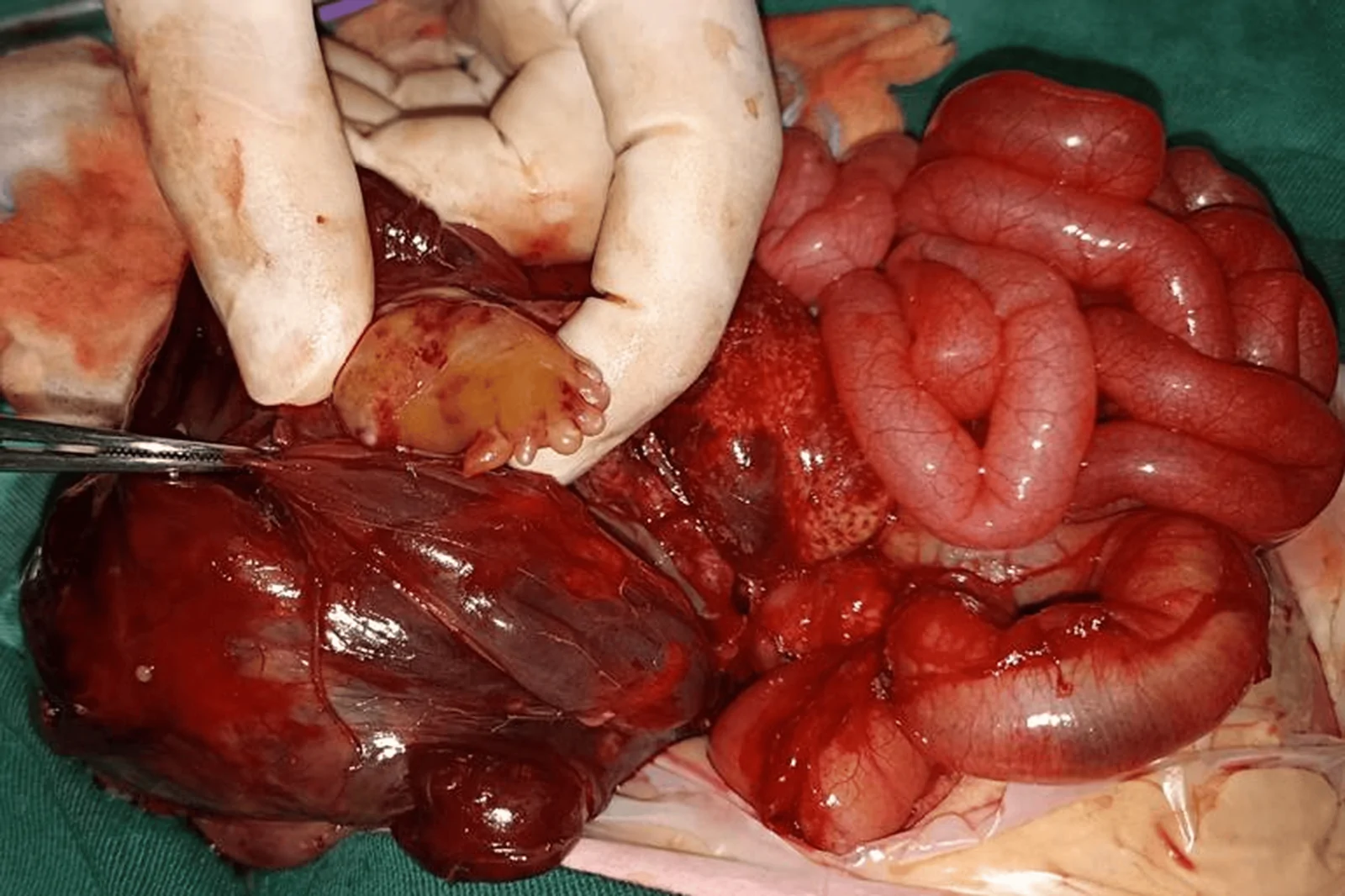
Intraoperative appearance of the tumor Intraoperative appearance of the tumor after recognition of pre-existing capsular disruption, showing an organized macroscopic structure resembling a hand. This finding raised the differential diagnosis of fetus in fetu.

At the beginning of the surgical exploration, the tumor capsule was already disrupted. Organized macroscopic structures resembling a hand and a foot were identified, raising the differential diagnosis of fetus in fetu. Because the lesion contained a cystic component and the capsule was disrupted, profuse irrigation of the operative field with saline solution was performed. No separate fluid sample was sent for histological analysis; however, the resected tumor specimen was submitted for complete histopathological examination.

The pancreatic head and body were preserved. Intraoperatively, the pancreatic tail was considered involved by the tumor and was therefore resected together with the lesion. The exact technique used for pancreatic transection was not specified in the available operative documentation and was therefore not retrospectively inferred.

During the dissection, the normal biliary anatomy was not clearly identifiable because of distortion of the regional structures. Given the complexity of the retroperitoneal dissection and concern for an unrecognized ductal injury, intraoperative cholangiography was performed as a patient-specific adjunct to delineate ductal anatomy and confirm biliary tract patency. The common bile duct was not documented as being directly compressed by the tumor. The available operative documentation does not indicate that a definite common bile duct or main pancreatic duct injury had been identified before cholangiography, nor does it document that the cholangiographic findings prompted an additional biliary or pancreatic procedure. Cholangiography demonstrated a patent biliary tract and visualization of the main pancreatic duct (Figure 4). This maneuver should therefore be interpreted as a patient-specific intraoperative adjunct rather than a routine requirement for neonatal retroperitoneal teratoma resection.

**Figure 4:**
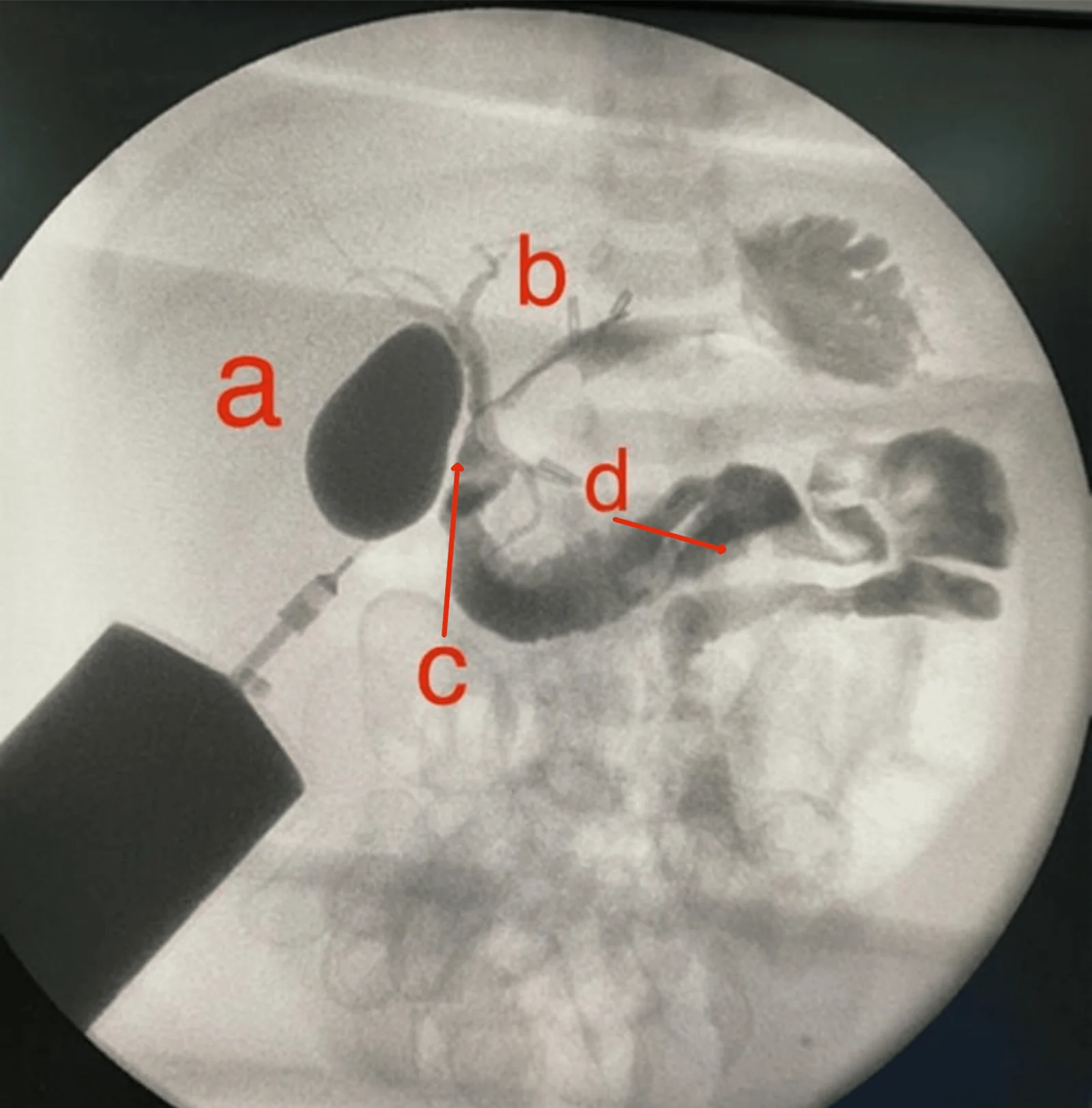
Intraoperative cholangiography Intraoperative cholangiogram demonstrating patency of the biliary tract and visualization of the main pancreatic duct. Labels: a, gallbladder; b, intrahepatic bile ducts; c, common bile duct; d, main pancreatic duct.

After tumor resection and pancreatic tail resection, a Penrose drain was placed toward the pancreatic surgical bed and the pelvic cul-de-sac to provide drainage and allow postoperative surveillance for possible pancreatic leakage or fluid collection.

The surgical specimen measured 11 × 8 × 5.5 cm and weighed 216 g. Gross examination demonstrated solid and cystic areas, cartilaginous foci, and a heterogeneous cut surface. Histopathological examination confirmed a prepubertal, solid-cystic, tridermic, grade 3 extragonadal immature teratoma without necrosis or elements of another germ cell tumor subtype. The pathology report described partial involvement of the pancreatic tail. However, the available pathology documentation did not specify whether pancreatic involvement was demonstrated microscopically at the pancreatic-tumor interface, did not provide a separate description of that interface, and did not report the status of the pancreatic resection margin. Therefore, the extent and microscopic nature of pancreatic involvement cannot be further defined from the available source records. No vertebral axis was identified (Figure 5).

**Figure 5:**
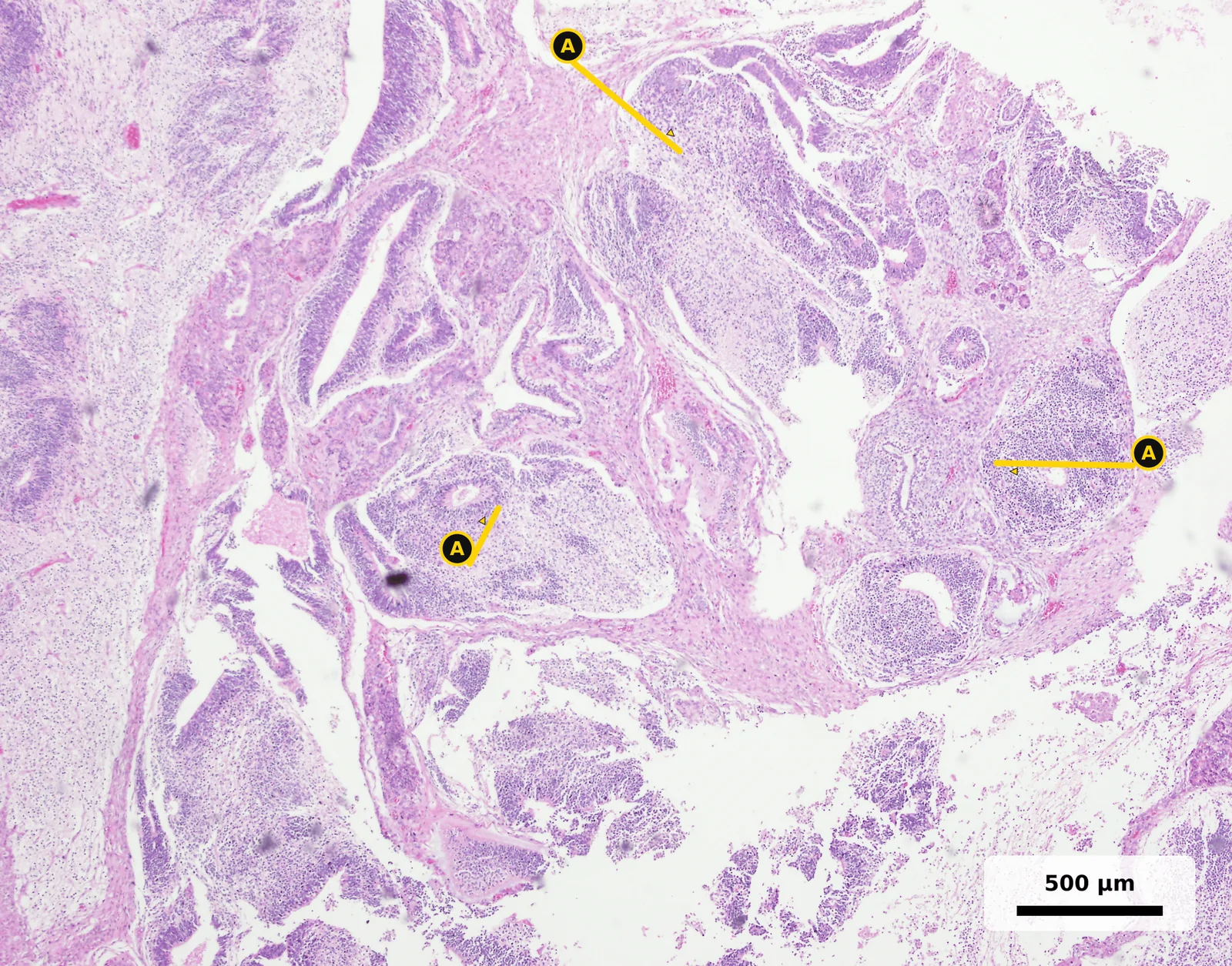
Histopathologic features of the immature teratoma Hematoxylin and eosin-stained section of the resected retroperitoneal tumor. Yellow arrows identify representative primitive neuroepithelial rosettes and tubules, consistent with an immature teratoma. Objective magnification: 4× (nominal optical magnification: 40× with a 10× eyepiece). Scale bar: 500 µm.

Postoperatively, the patient remained in the neonatal intensive care unit and required invasive mechanical ventilation for 10 days. Enteral feeding was initiated 72 hours after surgery. The Penrose drain initially produced approximately 80 mL, with progressive reduction in output over the following days. No clinically evident pancreatic fistula or intra-abdominal fluid collection was documented. The drain was removed on postoperative day 7 after satisfactory clinical evolution.

The total hospital stay was 41 days. A repeat alpha-fetoprotein (AFP) value before discharge was not available in the source record. Post-discharge oncology records were also not available among the source documents reviewed for this report. Consequently, subsequent oncology visits, serial age-adjusted AFP measurements, recurrence assessment, and the administration or absence of adjuvant treatment could not be confirmed.

Written informed consent for publication of the clinical information and images was obtained from the patient's legal representative.

## Discussion

Retroperitoneal teratomas are uncommon in children and particularly rare in neonates [1-11]. Large tumor size, proximity to solid organs, distorted retroperitoneal anatomy, and relationships with major vessels may increase the complexity of resection and perioperative risk [1,3,6].

Computed tomography and magnetic resonance imaging can define tumor composition, calcifications, and relationships with adjacent structures. In this patient, computed tomography demonstrated a solid-cystic mass with calcifications and close relationships with the pancreas and major vessels, information relevant for operative preparation [1,3,6].

Complete surgical resection is the principal treatment for pediatric immature teratomas when feasible [5,7,8,10,20]. In this case, the need to control pedicles arising from the aorta and draining toward the inferior vena cava, together with intraoperative assessment of pancreatic tail involvement, accounted for the technical difficulty. The pancreatic head and body were preserved, whereas the tail was resected. Importantly, although the pathology report described partial pancreatic tail involvement, the available pathology documentation did not separately characterize the pancreatic-tumor interface or the pancreatic resection margin. This prevents a more precise statement regarding the microscopic extent of pancreatic involvement.

AFP is physiologically elevated in neonates and decreases with postnatal age; therefore, a neonatal value should be interpreted using age-adjusted reference data rather than an adult threshold [19]. After successful resection of pediatric mature or immature teratomas, published pediatric experience and current National Cancer Institute (NCI) guidance support close clinical follow-up with serial serum AFP measurements. There is no single standardized schedule specifically for neonatal retroperitoneal immature teratoma; tumor markers are generally followed frequently for several years, and imaging is individualized according to clinical and biochemical findings [20]. In this patient, no repeat AFP value before discharge and no post-discharge oncology records were available, so the surveillance actually implemented, recurrence status, and any subsequent treatment cannot be determined.

The appearance of a hand and a foot prompted consideration of fetus in fetu [12-14]. Fetus in fetu is characterized by a higher degree of organotypic or axial organization, classically including a vertebral axis, whereas immature teratoma is defined histologically by immature tissue elements [12-15]. In this patient, the absence of a vertebral axis together with the histopathological finding of a grade 3 immature teratoma supported the final diagnosis of immature teratoma rather than fetus in fetu.

Intraoperative cholangiography was not used as a routine component of retroperitoneal teratoma surgery. It was performed in this patient because regional anatomy was distorted and the biliary tract could not be clearly delineated during dissection. The available operative record does not document a definite ductal injury before cholangiography or a subsequent additional biliary or pancreatic procedure prompted by the cholangiographic findings. Its role in this case was therefore anatomical clarification and confirmation of ductal patency in a specific complex operative setting.

The value of this case lies in the combination of neonatal presentation, grade 3 histologic immaturity, pancreatic tail resection, complex vascular relationships, and intraoperative morphology mimicking fetus in fetu. The report also illustrates the importance of separating intraoperative assessment from histologically documented findings when describing organ involvement.

This report has important limitations. The available pathology documentation did not provide a separate microscopic description of the pancreatic-tumor interface or the pancreatic resection margin. Long-term oncologic follow-up, serial age-adjusted AFP values, recurrence status, and information regarding any adjuvant treatment were not available in the source records. Therefore, the long-term oncologic outcome and the surveillance strategy actually implemented for this patient cannot be determined.

## Conclusions

Immature retroperitoneal teratoma should be considered in the differential diagnosis of a complex neonatal abdominal mass containing solid, cystic, and calcified components. Pre-operative imaging is essential to define the relationship of the tumor with major vessels and adjacent organs and to anticipate the technical complexity of surgical resection.

Intraoperative findings resembling fetal structures may suggest fetus in fetu; however, gross appearance alone is insufficient for diagnosis. Definitive distinction requires histopathological correlation, particularly assessment of axial or vertebral organization and the degree of tissue immaturity. When regional pancreaticobiliary anatomy is markedly distorted, intraoperative imaging may be used selectively as a patient-specific adjunct to clarify critical structures. Long-term outcome in this patient could not be assessed because post-discharge oncology records and serial AFP measurements were unavailable.
